# Public Health Workers and Vaccination Coverage in Eastern China: A Health Economic Analysis

**DOI:** 10.3390/ijerph110505555

**Published:** 2014-05-22

**Authors:** Yu Hu, Lingzhi Shen, Jing Guo, Shuyun Xie

**Affiliations:** Institute of Immunization and Prevention, Zhejiang Center for Disease Control and Prevention, Hangzhou 310051, China; E-Mails: lzhshen@cdc.zj.cn (L.S.); jguo@cdc.zj.cn (J.G.); shyxie@cdc.zj.cn (S.X.)

**Keywords:** public health worker, vaccination personnel, immunization, vaccination coverage, determinants

## Abstract

*Background*: Vaccine-preventable diseases cause more than one million deaths among children under 5 years of age every year. Public Health Workers (PHWs) are needed to provide immunization services, but the role of human resources for public health as a determinant of vaccination coverage at the population level has not been assessed in China. The objective of this study was to test whether PHW density was positively associated with childhood vaccination coverage in Zhejiang Province, East China. *Methods*: The vaccination coverage rates of Measles Containing Vaccine (MCV), Diphtheria, Tetanus and Pertussis combined vaccine (DTP), and Poliomyelitis Vaccine (PV) were chosen as the dependent variables. Vaccination coverage data of children aged 13–24 months for each county in Zhejiang Province were taken from the Zhejiang Immunization Information System (ZJIIS). Aggregate PHW density was an independent variable in one set of regressions, and Vaccine Personnel (VP) and other PHW densities were used separately in another set. Data on densities of PHW and VP were taken from a national investigation on EPI launched by Ministry of Health of China in 2013. We controlled other determinants that may influence the vaccination coverage like Gross Domestic Product (GDP) per person, proportion of migrant children aged <7 years, and land area. These data were taken from Zhejiang Provincial Bureau of Statistics and ZJIIS. *Results*: PHW density was significantly influence the coverage rates of MCV [Adjusted Odds Ratio(AOR) = 4.29], DTP_3_(AOR = 2.16), and PV_3_ (AOR = 3.30). However, when the effects of VPs and other PHWs were assessed separately, we found that VP density was significantly associated with coverage of all three vaccinations (MCV AOR = 7.05; DTP_3_ AOR = 1.82; PV_3_ AOR = 4.83), while other PHW density was not. Proportion of migrant children < 7 years and Land area were found as negative and significant determinants for vaccination coverage, while GDP per person had no effect on vaccination coverage. *Conclusions*: A higher density of PHWs (VP) would improve the availability of immunization services over time and space, which may increase the possibility of achieving a higher childhood vaccination coverage rate. It was indicated that the level of GDP per person had no association with the improved vaccination coverage after controlling for other potential factors. Our findings implicated that PHW density was a major constraint on immunization coverage in Zhejiang Province.

## 1. Introduction

Vaccine-preventable diseases are one of the most common and main causes of childhood death. According to the data released by World Health Organization (WHO) in 2008, almost 1.5 million of deaths among children < 5 years were due to contagious diseases that could be prevented by vaccination, which represents almost one fifth of the total mortality of children < 5 years globally [1]. More than 1 million these deaths could be prevented by using two vaccines alone: the Measles-Containing Vaccine (MCV) and the Diphtheria, Tetanus and Pertussis combined vaccine (DTP), which could potentially have prevented 540,000 and 496,000 deaths [1]. Vaccination coverage rate thus seems to be a key instrument in meeting the United Nation’s Millennium Development Goal of reducing under-5 mortality by two thirds in 2015 [2]. Indeed, the vaccination coverage rate of MCV for 1-year-old children is selected as one of the indicators to measure progress towards the Millennium Development Goal [3].

In this study we have focused on the role of public health workers (PHWs) in influencing vaccination coverage among children in Zhejiang Province in Eastern China. This study can be considered as a follow-up to previous studies that examined the role of human resources in affecting the health outcomes of maternal mortality, infant mortality, and under-5 mortality [4,5,6]. Other factors being equal, a higher density of PHW may be expected to result in a higher vaccination coverage. Apart from doing immunization, Chinese PHWs prepare vaccines for use, assess a child’s eligibility for a specific vaccine, rule out contradictions and document the vaccination record [7]. Additionally, they check that vaccines are transported and stored under appropriate conditions to ensure vaccine efficacy and safety, and they notify parents and other community members about the benefits and possible risks of immunization.

Notwithstanding the potential role of PHWs, the extent to which human resources for health influence vaccination is unknown, especially when other impact factors are taken into account. A previous study [8] used “regression analysis based on health workers density and health outcomes over the World” to conclude that “a density of about 1.5 workers per 1,000 population is associated with 80% coverage against MCV”. This analysis did not control for other determinants which could potentially affect the vaccination coverage, for example, socio-economic development level, proportion of migrant children, and scope of services. The density of health worker associated with 80% coverage in country will depend on the values of other determinants. There were numerous household- or individual- level studies that examined the determinants of childhood immunization coverage, but none include health workers as an explanatory variable [9,10,11,12,13,14]. 

Zhejiang is a developed province with a large population of 80 million people located in Eastern China [15]. Since 1978, Zhejiang Province initiated the EPI and has administered more than 20 million doses of vaccines each year according to the immunization schedule recommended by the Ministry of Health (MoH) of China [16]. The target of immunization coverage rate is set at 90% for all the vaccines, which is required to reach herd immunity and interrupt community transmission for all vaccine preventable disease [16]. In this study, we adopted multivariate regression analysis to evaluate the extent to which the density of PHW impacts the childhood vaccination coverage at the county level when we control for other determinants that seem likely to affect vaccination coverage. If PHW is found to matter independently of other determinants, we will assess the disaggregated categories of human resources associated with childhood vaccination coverage, for example, vaccination personnel (VP) and other PHW.

## 2. Methods

### 2.1. Choice of Dependent Variables

In our study, dependent variables were the vaccination coverage of three different vaccines: MCV, DTP, and Poliomyelitis Vaccine (PV). More precisely, the dependent variables measured the coverage with valid vaccination, namely, the proportion of children who receive all the required vaccine doses in the correct age windows. According to the vaccination schedule recommended by the China’s MoH, each child need to be vaccinated with the following vaccines by its first birthday [17]: a birth dose of Bacille Calmette-Guérin (BCG), three doses of PV, three doses of DTP, three doses of hepatitis B vaccine (Hep B), one dose of MCV, and one dose of Japanese Encephalitis Vaccine (JEV) (Table 1).

**Table 1:** Recommended childhood primary immunization schedule in China.

	Age								
	Birth	1 m	2m	3 m	4 m	5 m	6 m	7 m	8 m
BCG	Dose1								
Hep B	Dose1	Dose2					Dose3		
PV			Dose1	Dose2	Dose3				
DTP				Dose1	Dose2	Dose3			
MCV									Dose1
JEV									Dose1

There were two reasons for choosing MCV and DTP as dependent variables: first, these two vaccines are likely to prevent largest number of deaths [1]; second, they are used to monitor childhood vaccination coverage rates and trends [18]. We used MCV and DTP coverage as separate dependent variables to allow for the possibility that the association between density of PHW and these two vaccination variables may be different. To be counted as fully vaccinated against measles, a child needs to have be vaccinated only one dose of MCV by its first birthday. However, to be counted as fully vaccinated against diphtheria, tetanus and pertussis, a child needs to have been with vaccinated three doses of DTP by his/her first birthday (we denoted completion of DTP as DTP_3_). Obviously, for a child to be fully vaccinated against diphtheria, tetanus, and pertussis, the contact between the PHW and the child needs to occur more frequently than for the child to be vaccinated against measles. Another difference between these two vaccines is that MCV, unlike DTP, is sometimes delivered in short-term campaigns as well as through routine immunization according to the recommendation of China’s MoH [19].

There were also two reasons for choosing coverage of PV as a further dependent variable: first, unlike MCV, PV needs to be vaccinated in three doses, the completion of which was denoted as PV_3_; second, unlike DTP, PV is delivered in China not only through routine immunization but also through vaccination campaigns or supplementary immunization activities (SIAs) such as “national immunization days” [20]. According to the Standard Guideline on Immunization issued by MoH of China in 2004 [21], the SIA doses are recorded in the immunization card separately and not included in the coverage estimates for routine immunization program. In these SIAs, the full range of available human resources from unskilled volunteers to highly skilled PHWs, both inside and outside the health sector are temporarily mobilized as vaccinators [22]. Since PV_3_ vaccination coverage may be impacted by an informal workforce, the association between human resources for public health and vaccination coverage of PV_3_ may be different from that of either MCV or DTP_3_.

### 2.2. Choice of Independent Variables

First, as an aggregate method, we included the sum of PHWs: full-time VP and part-time other PHWs. These PHWs are trained to administer all vaccines used as dependent variables. Second, in order to assess the effect of different types of PHW on vaccination coverage, we split the aggregate variable of PHWs into two categories: (1) VPs who take the vaccination as a full-time job and (2) other PHWs who take the vaccination as part-time job or act as a unskilled volunteer in vaccination campaign. Third, these variables were collected for each county and used in density form: PHW, VP, and other PHW densities were the number of PHW, VP or other PHW per 10,000 population. In order to account for the socioeconomic development influence of vaccination coverage, we included Gross Domestic Product (GDP) per head of population for each county as a general resources variable. We also included proportion of migrant children aged < 7 years (the number of migrant children aged < 7 years divided by the total number of children aged < 7 years) for each county as an independent variable because the association between the proportion of migrant children and the vaccination coverage was well reported by previous studies [23,24,25]. Finally, we included land area for each county as an independent variable. For any given density of PHWs, we expect a county with a larger land area to have lower vaccination coverage: generally, the distance between health workers and children is likely to be greater and the accessibility of immunization service is likely to be poorer.

### 2.3. Data Sources

Data on vaccination coverage were taken from the Zhejiang Immunization Information System (ZJIIS), which was an electronic immunization record system established in 2004. All the immunization clinics were enrolled in the ZJIIS since 2004. All the children including migrant children are registered in ZJIIS at their first point of contact with immunization clinics and have been given a unique identification number in the ZJIIS [26]. Children born from 31 December 2011 to 1 December 2012 registered in ZJIIS were enrolled in our study. Immunization records of target children were queried from the provincial database of ZJIIS on 31 December 2013. The vaccination coverage rate of children was calculated using the number of target children immunized with the vaccines of interest as the numerator, and the total number of ZJIIS-registered target children as the denominator. All three dependent variables were expressed as the percentage of children who had received specific vaccines at any time before their first birthday. Our data on densities of PHW and VP of each counties were taken from a national investigation on EPI of China, which was launched by MoH of China in September 2013. This source was considered as the most reliable and latest dataset on PHW and PV currently available. These data were collected from health bureaus of all of the 90 counties in Zhejiang province and were well cross-checked with alternative records like censuses, labor force surveys or recent publications. The data of other independent variables such as total population of each county, GDP per person, and land area (defined as a county’s total area, excluding area under inland water bodies) were obtained from the Zhejiang Provincial Bureau of Statistics [15]. We also obtained the proportion of might children < 7 years of age from ZJIIS through dividing the number of migrant children by the total number of children registered in ZJIIS.

### 2.4. Data Analysis

Description analysis was used to present the vaccination coverage for each of the three vaccines, PHW/VP/other PHW densities, and other independent variables for all of the 90 counties in Zhejiang province. Vaccination coverage rate of each vaccine was dichotomized as “<90% or ≥90%”. Single-level logistic regression model was adopted to evaluate the association between vaccination coverage and human resources of public health and Adjusted Odds Ratio (AOR) with 95% *CI* was calculated. We reported two sets of logistic regression: first, we regressed the level of vaccination coverage against aggregate PHW density, while controlling for GDP per head of population, proportion of migrant children aged < 7 years and land area. The second set of regressions mimicked the first set, but with PHW density disaggregated into VP density and other PHW density as separate independent variables. All the analysis above applied Statistics Package for Social Science (SPSS) software, version 13.0.

## 3. Results

Table 2 presents the mean and Standard Deviation (SD) and range of all dependent and independent variables in natural units. Eighteen out of 90 (20.00%) counties’ coverage rates of MCV were lower than 90%, and 25 (27.78%) counties’ coverage rates of PV_3_ and 30 (33.33%) counties’ coverage rates of DTP_3_ were lower than 90%. 

The density of PHW in aggregate terms was positively and significantly associated with vaccination coverage of all three vaccines (MCV: AOR = 4.29; DTP_3_: AOR = 2.16; PV_3_: AOR = 3.30) after controlling other determinants of vaccination coverage. When VP and other PHW densities entered separately in the logistic regression models, the VP density was positively and significantly associated with vaccination coverage of all three vaccines (MCV: AOR = 7.05; DTP_3_: AOR = 3.13; PV_3_: AOR = 4.83) while other PHW was not (95% CIs of all three vaccines included a null value). Proportion of migrant children < 7 years and land area became negative and significant determinants for all of three vaccines when aggregate PHW density or VP density were set as independent variable separately. The AOR for proportion of migrant children < 7 years ranged from 0.49 to 0.68 while that for land area ranged from 0.47 to 0.72. GDP per person was not a significant predictor of coverage of any vaccines when aggregate PHW density, the VP density, or other PHW density was set as independent variable separately (Table 3).

**Table 2:** Description analysis of dependent and independent variables.

Variables	Mean(SD)	Range
MCV coverage (%) ^*^	93.81% (4.92%)	86.37%–98.26%
DTP_3_ coverage (%) ^*^	87.14% (6.32%)	76.11%–96.84%
PV_3_ coverage (%) ^*^	90.47% (6.05%)	82.30%–97.41%
GDP per person (CNY)	68,593.06 (17,830.86)	49,838.21–94,791.18
Proportion of migrant children < 7 years (%)	51.58% (18.36%)	11.63%–75.20%
Land area(Km^2^)	1,131.06 (1,178.75)	67.95–4,427.15
PHW density (per 10,000 population)	1.62 (1.74)	0.29–6.91
VP density (per 10,000 population)	0.13 (0.28)	0.03–3.74
Other PHW density (per 10,000 population)	1.48 (1.61)	0.21–4.68

Note: ^*^ percentage of children aged 13–24 months (born from 31 December 2011 to 1 December 2012) registered in ZJIIS.

## 4. Discussion

Our study found that PHW density was a significant determinant of vaccination coverage. This link may be self-evident in individual level because vaccinations are administrated in an interaction between a health-care provider and a person seeking immunization service. However, this association between human resources of public health and vaccination coverage is less evident at the population level. The significant association between human resources of public health and vaccination coverage at population observed in our study may be due to the following reasons: PHWs on average spend only a small proportion of their working time administrating vaccines in China [27]. However, since the demand of vaccination is not concentrated in time or place, PHWs need to be available over time and space to meet the rising demand. Parents would need to have their children immunized in moving windows over time as children are born throughout the year. Thus PHWs are required to vaccinate uniformly over the year, even if most of their time may be spent providing other public health services.

Other factors being equal, the higher the density of PHW, the higher will be the availability of immunization service over time and space, and it is more likely that the immunization will take place. Although the higher availability of vaccination services made possible by a higher density of PHW, there are other pathways that could contribute to the association at the population level. PHW may increase the demand for vaccinations by educating caregivers of children on vaccination and raising overall health awareness in the community. Besides, they may spend some of their working time ensuring that vaccines are available continually and stored appropriately. Finally, they could contribute to improved vaccination coverage by training the unskilled volunteers who administer vaccinations in the case of the polio or measles specific SIAs. Further studies are needed to investigate the different pathways between density of PHW and improved vaccination coverage. 

**Table 3:** Multivariable analysis on assessing the association between vaccination coverage and human resources of public health.

Independent Variables	Regression with Aggregate PHW Density as an Independent Variable AOR(95% *CI*)			Regression with VP Density as an Independent Variable AOR(95% *CI*)			Regression with other PHW Density as an Independent Variable AOR(95% *CI*)		
	MCV Coverage	DTP_3_ Coverage	PV_3_ Coverage	MCV Coverage	DTP_3_ Coverage	PV_3_ Coverage	MCV Coverage	DTP_3_ Coverage	PV_3_ Coverage
GDP per person	1.62 (0.84–3.11)	1.72 (0.75–2.53)	2.13 (0.76–5.52)	2.02 (0.59–4.75)	1.59 (0.66–3.94)	1.84 (0.82–4.95)	1.13 (0.49–2.36)	3.21 (0.82–13.40)	2.58 (0.58–7.35)
Proportion of migrant children < 7 years	0.49 (0.31–0.84)	0.63 (0.32–0.92)	0.68 (0.22–0.89)	0.52 (0.25–0.87)	0.72 (0.49–0.88)	0.47 (0.28–0.73)	0.58 (0.33–0.74)	0.81 (0.36–2.28)	0.64 (0.35–0.81)
Land area	0.63 (0.32–2.95)	0.58 (0.33–0.90)	0.49 (0.25–0.88)	0.58 (0.39–0.86)	0.62 (0.41–0.85)	0.47 (0.37–0.73)	0.73 (0.25–0.81)	0.33 (0.21–0.69)	0.50 (0.37–0.91)
PHW density	4.29 (2.06–15.84)	2.16 (1.26–5.29)	3.30 (1.83–6.77)	-	-	-	-	-	-
VP density	-	-	-	7.05 (3.97–20.14)	3.13 (1.82–7.18)	4.83 (2.14–16.85)	-	-	-
Other PHW density	-	-	-	-	-	-	2.19 (0.74–6.02)	1.74 (0.70–3.35)	1.88 (0.66–3.96)

Our findings showed that VP density was a significant determinant of vaccination coverage, while that other PHW density was not, could be due to the practice in most conditions of VPs, but not other PHWs, administering vaccines. Other PHWs obviously have the capacity and are license to administrate vaccines and ancillary tasks, but they may find other health service they can do are more pressing, professional or financially rewarding than vaccination. Of course, VPs may not be capable of delivering those health services. The opportunity cost of other PHW’s time could be too high to administer vaccines. As a result, other PHWs may actually give vaccinations for none or a negligible fraction of their working time. Hence, an increase in other PHW density will not lead to a significant change in vaccination coverage. By contrast, VPs could find that using their working time for educating caregivers, participating in SIAs, and training volunteers are valuable and meaningful. 

In addition to the finding that VP density was associated with the vaccination coverage of all three vaccines, we found that the coefficients were similar in size. The robustness was found despite disparities among the vaccines in number of doses required for valid vaccination, in modes of delivery (oral *versus* injected), and in use of SIAs. Whatever the accurate role of VP in these three different regimens (e.g., practicing routine immunization, training or supervising unskilled volunteers), it was assumed that VP was a sufficient and prerequisite human resource to achieve a optimistic coverage.

We found that GDP per person was not associated with coverage rates of all three vaccines whether aggregate the PHW as an independent variable or not. Social-economic development is significant in reducing maternal, infant, and under-5 mortality [28], while it is reasonable that it should not impact vaccination coverage. Consisted with previous studies [6], many of the important determinants captured by social-economic development status that influence mortality rates are unlikely to impact vaccination coverage, for example, nutrition, safe water, and sanitation. Generally, the budget for vaccine is covered and allocated at national level, while budget for immunization practice, cold chain maintenance, staff training, surveillance, and health education is covered and allocated at county level in China. Although GDP does include some elements that are expected to influence vaccination coverage, those spending is only a small fraction of GDP. Moreover, since this fraction is varied across counties, cross-county variations in GDP per person will not reflect actual difference in expenditure on these items. It is still a coarse index of vaccine-related expenditure. In this study, we only assessed the association between GDP per person and vaccination coverage at county level in Zhejiang Province due to the data limitation. There was a possibility that GDP per person would be significantly associated with coverage rate if it was extended to the whole China and further studies are needed.

The proportion of migrant children aged < 7 years for each county was found to be a negative factor associated with coverage of all three vaccines. The results of this study probably indicated that the awareness of and access to the vaccination service for parents of migrant children may be different from that for parents of resident children. Moreover, since the migrant people have a poor living condition, they may spend more time and effort to adapt the new sociocultural environment, they are not likely to consider the childhood vaccination as a higher priority [29]. We found that proportion of migrant children aged < 7 years was more significant and had larger ORs for DTP_3_ than for MCV or PV_3_. As we all know, MCV needs to be vaccinated only once, whereas DTP_3_ and PV_3_ need to be vaccinated three times with an interval of one month according to the immunization schedule recommended by the China’s MoH [17]. A one-time visit to a health center may be motivated by purposes other than vaccination, but subsequent visits at predetermined times require advance planning, for which the education level of caregivers would be helpful. Although the PV, as well as DTP, needs to be administered three times for a child to be fully immunized, poliomyelitis, unlike diphtheria, tetanus, and pertussis, has focused on a global eradication objective [30]. These activities are likely to diminish the importance of literacy and health awareness of migrant caregivers in influencing vaccine coverage. 

Finally, we found that land area of each county was negatively associated with vaccination coverage, and the ORs were significant for DTP_3_ and PV_3_ but not for MCV. Generally, with a larger land area, a given number of PHWs per person will be spread more thinly over space, and hence the geographical availability of immunization services will be smaller. A decrease in spatial availability adds to the difficulty, including time and travel costs, of getting a child to a health center for vaccination, or *vice versa*. For a child to be fully vaccinated for DTP and PV, these costs are needed three times, whereas in the case of MCV they occur only once.

## 5. Conclusions

The findings of our study implicated the importance of PHW for vaccination coverage among children in Zhejiang Province at a county-level. They also provided indirect support for the GAVI Alliance’s assertion that human resources may be “a ‘major barrier’ to improving immunization coverage” [31]. If we increase PHW and VP densities, we will achieve significantly higher vaccination coverage. Larger increases will be needed to achieve the same level of coverage in areas with a larger land area compared with a smaller one. Furthermore, migrant children were proved as a vulnerable group for vaccination and vaccine-preventable diseases and the level of GDP per person had no independent effect on vaccination coverage after controlling for other determinants in this study. At last, we suggest that government or other non-government organization that interested in expanded immunization program to reduce infant and child mortality should, apart from vaccine-related assistance, focus their efforts on human resources for health.
